# Micrometre-Scale Surface Partitioning and Morphological Anisotropy in Reacted Bioactive Glass Quantified by SEM–EDS

**DOI:** 10.3390/ma19204317

**Published:** 2026-10-14

**Authors:** Helena Cristina Vasconcelos, Maria Gabriela Meirelles

**Affiliations:** 1 Faculty of Science and Technology, University of the Azores, S. Miguel, Azores, 9500-321 Ponta Delgada, Portugal; maria.gf.meirelles@uac.pt; 2 Laboratory of Instrumentation, Biomedical Engineering and Radiation Physics (LIBPhys, UNL), Department of Physics, NOVA School of Science and Technology, 2829-516 Caparica, Portugal; 3 Research Institute of Marine Sciences, University of the Azores (OKEANOS), Faial, Azores, 9901-862 Horta, Portugal

**Keywords:** bioactive glass, simulated body fluid, SEM–EDS, computational image analysis, spatial association, morphological anisotropy

## Abstract

Reacted bioactive-glass surfaces often contain Ca–P-rich deposits beside Si-rich regions, but qualitative overlap in SEM–EDS maps cannot establish whether Ca and P are spatially associated or merely co-occur because clustered maps occupy finite areas. Here, one archived EDS field and one separate SEM field from each of two glasses of the same nominal composition, prepared with geothermal water (77S-G) or distilled/deionised water (77S-R) and reacted for 216 h in simulated body fluid, were analysed computationally. The EDS inputs were RGB JPEG occurrence maps rather than raw-count maps. Binary elemental supports were evaluated over 16 segmentation settings, and observed overlap was compared with 47,879 non-zero toroidal translations. Ca and P remained associated throughout the sweep (median zAB = 3.31 for 77S-G and 2.48 for 77S-R) and sensitivity checks. In 77S-R, Ca–Si separation was stable, while P–Si remained negative. A local Ca–P-rich volume in 77S-G had Ca/P = 0.93 and was not assigned to hydroxyapatite. Effective lateral correlation scales were 0.40–4.56 µm, and the analysed 77S-G SEM field showed greater directional coherence over 0.2–1.2 µm. These are image-derived field-level descriptors; they do not identify a crystalline phase, establish a causal effect of synthesis water, or estimate specimen-wide properties.

## 1. Introduction

When a silicate bioactive glass is immersed in simulated body fluid (SBF), its surface evolves through ion exchange, network dissolution, silica reorganisation, and the accumulation of calcium- and phosphate-containing material [1,2]. These processes occur within the same interfacial region and produce a surface that may vary considerably over micrometre distances. The effect is particularly evident in powders, where the particle curvature, exposed porosity, and access to the surrounding solution vary among particles. Silica-rich regions can coexist with deposits enriched in calcium and phosphorus, while the surface morphology may range from compact areas to plate-like or acicular aggregates. A recent examination of reacted 45S5 glass by electron microscopy and spatially resolved surface analysis has confirmed the coexistence of Ca–P-rich material, Si-rich altered regions, and intervening Ca-rich zones [3].

The spatial arrangement of these features provides information that is lost when the elemental composition is reduced to an average value. The association between calcium and phosphorus can indicate the development of a common surface deposit, while their spatial separation from silicon locates that deposit relative to the reacted glass surface. The dimensions, continuity, and directional organisation of the resulting features also describe the physical structure of the interface. Surfaces with similar overall elemental contents may thus display markedly different spatial arrangements.

SEM–EDS provides access to this heterogeneity by combining surface morphology with spatially resolved elemental signals. In studies of reacted bioactive glasses, Ca, P, and Si distributions are usually examined through juxtaposed elemental maps, selected local spectra, and composition tables [1,2,3]. Quantitative approaches to SEM–EDS data have addressed the analytical reliability and elemental colocation in other material systems [4,5,6]. Image analysis has also been used to quantify morphological changes in thermally treated bioactive glass after SBF exposure, including changes in particle dimensions and elongation [7]. For reacted bioactive-glass surfaces, however, it remains unclear whether apparent Ca–P colocation reflects their relative placement or simply the occupied fraction and internal clustering of each displayed map.

Here, elemental co-occurrence means only that two signals are present within the same mapped field; spatial association means that their measured relative placement yields overlap in the tail of a field-specific translation reference; and phase identification requires independent chemical or crystallographic evidence. The primary contribution is therefore methodological: a field-preserving workflow for extracting the spatial association, lateral persistence, and directional coherence from archived SEM–EDS image records. The 77S-G and 77S-R fields serve as applications of the workflow rather than as replicated estimates of material-wide behaviour.

Two questions guide the analysis. First, does the measured Ca–P overlap exceed the overlap obtained when the original elemental supports retain their geometry but their relative positions are changed? Second, what lateral persistence is present in the elemental patterns, and how much directional order is present in the separately acquired SEM texture? To answer the first question, binary supports obtained over 16 colour-extraction settings are compared with every non-zero toroidal translation of the measured fields. Robustness is then examined through image quadrants, controlled registration shifts, crop margins, and an alternative overlap coefficient.

The second question is addressed with two-point correlation functions for the elemental supports and a multiscale structure tensor for the SEM texture. These measurements provide effective correlation lengths for the mapped elemental patterns and directional-coherence values for the high-resolution morphology over defined physical scales. Taken together, they provide complementary field-level descriptions of elemental placement, lateral persistence, and morphological anisotropy without assuming pixel-wise registration between the EDS maps and the high-resolution SEM images.

Accordingly, the study treats the microscopy records as a combined experimental–computational characterisation problem. The objective is not to infer phase identity from displayed elemental maps, but to quantify spatial information that conventional visual comparison leaves ambiguous. Exhaustive translation controls and sensitivity analyses test relative elemental placement, two-point correlations define lateral persistence, and a multiscale structure tensor quantifies directional order in the separately acquired SEM texture. By keeping these descriptors distinct, the workflow provides a transferable quantitative framework for heterogeneous bioactive-glass reaction surfaces while retaining the evidential limits of the underlying microscopy records.

## 2. Materials and Methods

### 2.1. Glasses and SBF Exposure

The two glass powders had the nominal composition 77SiO_2_–14CaO–9P_2_O_5_ (mol%) and were prepared using a sol–gel route based on the previously reported mineral-water-assisted procedure [8]. The sample prepared with mineralised geothermal water is designated 77S-G, and the reference sample prepared with distilled and deionised water is designated 77S-R. In related sol–gel bioactive-glass systems, Azorean geothermal waters used as synthesis media have been shown by XPS to modify the surface chemical environment and silicate-network connectivity [9]. These samples correspond to the original laboratory codes A77S and P77S, respectively. The chemical composition and physicochemical properties of the synthesis water are not input variables in the present image-analysis workflow, which operates exclusively on the archived microscopy records. Briefly, the reported mineral-water-assisted sol–gel preparation involved precursor hydrolysis and condensation in the selected aqueous medium to form the gel, followed by drying and the subsequent thermal treatment; full preparative details are given in Ref. [8].

After drying, the powders were thermally treated at 700 °C for 2 h. For SBF exposure, 100 mg of each powder was immersed in 10 mL of corrected simulated body fluid (c-SBF; pH 7.4), prepared according to the Kokubo–Takadama procedure [10]. The suspensions were maintained at 36.5 °C for 216 h. The recovered powders were drained and stored over silica gel until SEM–EDS examination.

The nominal c-SBF composition and preparation followed the cited Kokubo–Takadama protocol [10]: 142.0 mM Na^+^, 5.0 mM K^+^, 1.5 mM Mg^2+^, 2.5 mM Ca^2+^, 147.8 mM Cl^−^, 4.2 mM HCO_3_^−^, 1.0 mM HPO_4_^2−^ and 0.5 mM SO_4_^2−^, buffered with Tris and adjusted to pH 7.40 at 36.5 °C with HCl. The surviving laboratory record does not state whether the suspensions were static or agitated, whether c-SBF was refreshed during the 216 h exposure, or the precise rinsing and post-immersion drying steps beyond draining and storage over silica gel. It also does not establish whether the immersion was performed once or with independent replicates. The archived microscopy dataset represents one analysed EDS field and one separately acquired 1500× SEM field per material. Supplier, grade and reagent-purity details for the c-SBF preparation are not retained in the surviving laboratory record and have therefore not been reconstructed.

### 2.2. SEM–EDS Data

The available records comprise secondary-electron images, elemental-distribution maps, and local EDS spectra. The SEM–EDS measurements analysed in this study were performed at the Environmental Chemistry and Life Sciences Laboratories, Center of Innovation and Entrepreneurship, Fall River, MA, USA. The preserved image files indicate an accelerating voltage of 20 kV. For the 1500× high-resolution fields used in the comparative morphology analysis, the microscope model and working distance were not retained. Detector model, acquisition time, and count-rate information were also absent from the preserved records. Before examination, the powders were coated with a thin conductive Au layer. Gold was not included in the archived normalised EDS results. The original instrument exports retained in the study record document the nominal magnification, scale bars, accelerating voltage, and acquisition dates. No microscope model, working distance, or EDS-detector specification was assigned to an analysed field unless supported by its own preserved record. These missing parameters limit retrospective instrumental reproducibility and preclude raw-count reanalysis, but they do not alter the pixel coordinates of the mutually registered channel exports. For the mapped Si, P, and Ca signals, 20 kV provides sufficient overvoltage for the relevant K-shell X-ray lines; increasing the voltage to 30 kV would not be inherently superior for spatial mapping because it would also enlarge the electron interaction volume and could reduce spatial localisation [4].

**Figure 1:**
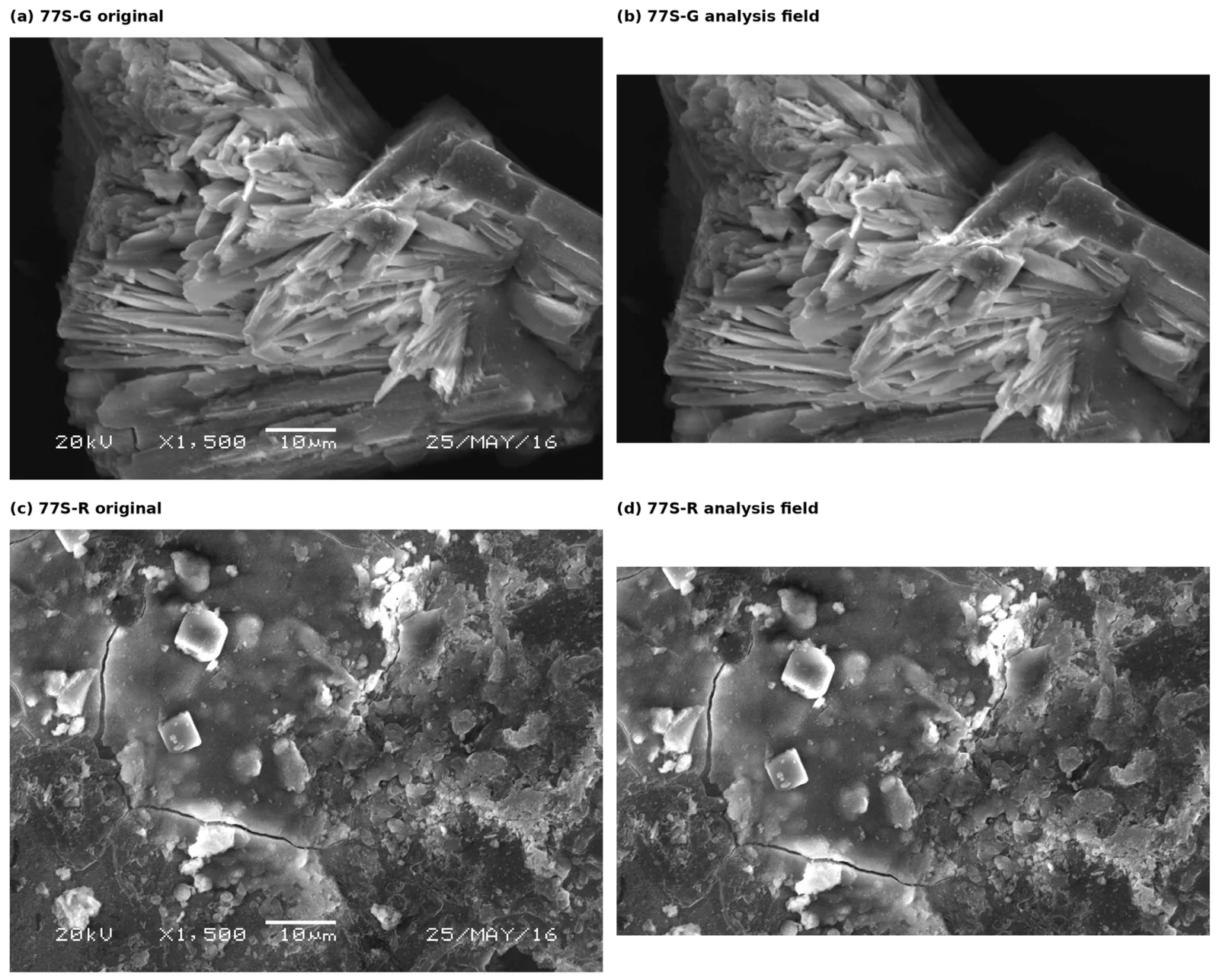
Original SEM records and corresponding analysis fields for the reacted surfaces: (**a**) original uncropped 77S-G instrument export and (**b**) the corresponding upper specimen field used for morphological analysis; and (**c**) original uncropped 77S-R instrument export and (**d**) the corresponding analysis field. The original exports retain the instrument-generated annotations, including 20 kV, 1500× magnification, 10 µm scale bars, and acquisition-date information. For computational texture analysis, the lower annotation-bearing region was excluded so that instrument text and scale-bar graphics did not enter the image gradients. The retained specimen field was not rescaled, composited, or internally edited. These SEM morphology fields are separate acquisitions from the EDS mapping fields in Figure 2.

**Figure 2:**
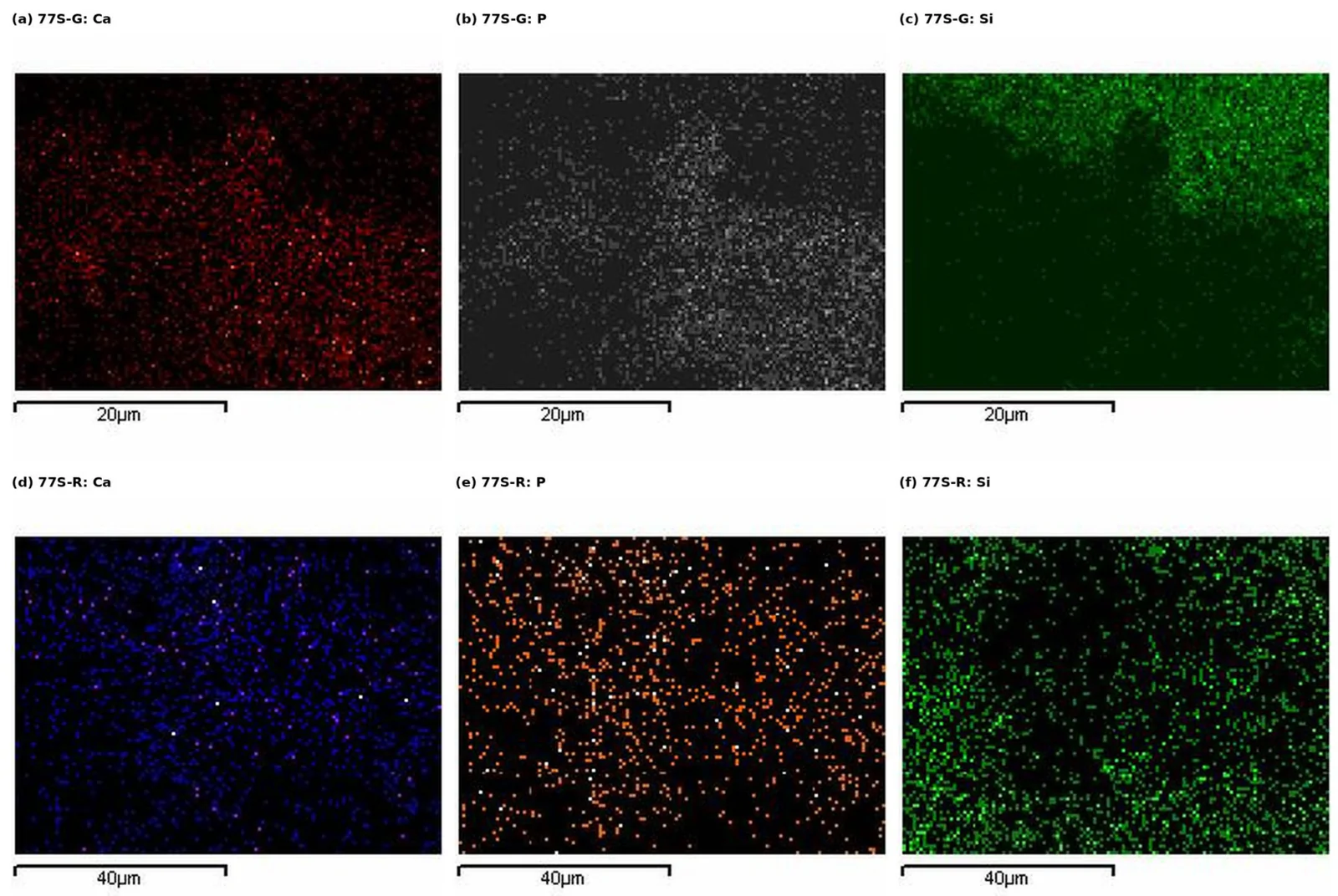
Original colour-coded elemental-map exports used as the source records for the spatial analysis: (**a**–**c**) Ca, P, and Si distributions in the 77S-G acquisition; and (**d**–**f**) Ca, P, and Si distributions in the 77S-R acquisition. Maps within each row originate from the same EDS acquisition and retain their original pixel registration and instrument-export scale-bar information. The displayed colours identify the elemental channels and do not represent concentration. The registered analysis field was obtained by applying the identical documented crop to every channel within an acquisition; the corresponding nominal segmented supports are shown in Figure S2.

The elemental maps were preserved as 8-bit RGB JPEG images. Coloured pixels represent the displayed occurrence of each element, but do not retain the underlying X-ray counts. The Ca, P, and Si channels belonging to each EDS acquisition have identical image dimensions and share the same pixel coordinate system. The spatial analysis was therefore performed on the positions and geometries of the displayed elemental regions and required registration among the elemental channels only. The high-resolution SEM images used for morphological analysis were treated separately. In particular, the high-resolution SEM fields shown in Figure 1 are separate acquisitions from the EDS mapping fields shown in Figure 2; no pixel-wise registration between these two data types was assumed. The local EDS spectra were used as semiquantitative measurements of the selected interaction volumes, with elemental concentrations normalised by the original acquisition software. The two local spectra from 77S-G share the same SEM field thumbnail, although their exact analysis coordinates were not retained in the exported reports. Acquisition time and count rate cannot be reconstructed from the surviving exports. Consequently, the sensitivity analyses assess the stability of the image-derived geometry to extraction and registration choices; they are not presented as validation against unavailable quantitative X-ray-count data.

The acquisition software generated separate colour-coded display maps for the selected elements, and the surviving files are the resulting JPEG exports. A common palette was not used across the two acquisitions: Ca, P, and Si were red, grey, and green in 77S-G, whereas they were blue, red, and green in 77S-R. Each map was therefore segmented according to its recorded channel colour. The files do not show whether JPEG compression was introduced during acquisition or during a subsequent export step. RGB values were treated only as display encodings of elemental occurrence and not as quantitative X-ray intensity or concentration.

### 2.3. Extraction and Spatial Association

The image region used for analysis was cropped identically from all elemental channels within each acquisition. Using zero-based indexing, rows 23–212 and columns 2–253 were retained from each 256 × 256 pixel export. This deterministic crop excluded the annotation-bearing margins, element labels, and scale-bar graphics from the computational field and produced mutually registered elemental fields of 252 × 190 pixels. The original 125-pixel scale bars represented 20 µm for 77S-G and 40 µm for 77S-R, corresponding to pixel sizes of 0.16 and 0.32 µm, respectively. The same coordinates were applied to every elemental channel in a given acquisition; within the retained analysis field, no specimen pixel was selectively deleted, repositioned, rescaled, or composited. Figure 2 now shows the uncropped source exports, while the corresponding nominal derived masks are shown in Figure S2.

Binary supports were extracted from the red, green, blue, and grey occurrence maps using the RGB channel values R, G, and B, expressed on a scale from 0 to 255. For a brightness threshold T and a channel-dominance factor d, the colour-selection criteria were

$$M_{\mathrm{red}}=\left(R>T\right)\cap \left(R>dG+5\right)\cap \left(R>dB+5\right),$$

$$M_{\mathrm{green}}=\left(G>T\right)\cap \left(G>dR+5\right)\cap \left[G>\max\left(1.08,d-0.07\right)B+5\right],$$

and

$$M_{\mathrm{blue}}=\left(B>T\right)\cap \left[B>\max\left(1.08,d-0.04\right)R+4\right]\cap \left[B>\max\left(1.08,d-0.04\right)G+4\right].$$

Grey occurrence marks were selected from their mean luminance and RGB chroma:

$$M_{\mathrm{grey}}=\left(\frac{R+G+B}{3}>T+7\right)\cap \left[\max\left(R,G,B\right)-\min\left(R,G,B\right)<24\right].$$

In 77S-G, the Ca, P, and Si supports were extracted from the red, grey, and green maps, respectively. In 77S-R, Ca, P, and Si corresponded to the blue, red, and green maps; the grey map represented O. A binary opening with a 2 × 2 pixel structuring element was applied to each support to remove isolated marks produced by JPEG compression. A nominal setting of T = 38 and d = 1.22, corresponding to the predefined default of the extraction routine, was used for the single-field summaries and robustness checks. Dependence on segmentation was evaluated separately over all 16 combinations of T = 28, 38, 48, and 58 with d = 1.12, 1.18, 1.24, and 1.30, which bracket the nominal dominance setting. No extraction setting was selected on the basis of the resulting association score. The nominal binary supports are shown in Figure S2.

For a pair of elemental supports A and B, an eight-connected binary dilation was applied with tolerance radii r=0, 1, 2, and 3 pixels. The radius was applied independently to each support and represents a morphological matching tolerance, not a direct centre-to-centre separation distance. The dilated supports are denoted by

$$A_{r}=D_{r}\left(A\right),\;\;B_{r}=D_{r}\left(B\right).$$

Because both supports are dilated, two original pixels can contribute to overlap even when separated by up to 2r pixels in an idealised one-dimensional example. Thus, r = 2 denotes a two-pixel expansion of each mask; the corresponding 0.32 µm (77S-G) and 0.64 µm (77S-R) values are per-support dilation radii and are not direct Ca–P separation distances. The effective match also depends on the two-dimensional feature geometry.

Their spatial overlap was measured using the Jaccard index:

(1) $$J_{AB}\left(r\right)=\frac{\left|A_{r}\cap B_{r}\right|}{\left|A_{r}\cup B_{r}\right|}.$$

The observed value was compared with the complete reference distribution obtained by translating support B relative to support A over every possible periodic displacement except the observed zero shift. For each displacement vector Δ, the translated overlap was calculated from the same Jaccard expression.

(2) $$J_{AB}^{0}\left(r;\Delta \right)=\frac{\left|A_{r}\cap T_{\Delta }\left(B_{r}\right)\right|}{\left|A_{r}\cup T_{\Delta }\left(B_{r}\right)\right|}$$

Periodic wrapping retains the occupied area, domain geometry, and autocorrelation of each support while changing the placement of B relative to A. A 252 × 190 field contains 47,880 toroidal positions; exclusion of the zero shift leaves 47,879 reference translations for every measured support pair and segmentation setting [11].

The exhaustive calculation was applied at dilation radii of 0, 1, 2, and 3 pixels. The nominal comparison used brightness 38 and dominance 1.22, while the full 16-setting sweep measured sensitivity to colour extraction. Directional empirical probabilities were obtained from the proportions of translated overlaps at least as large as, or no larger than, the observed overlap. The two-sided empirical probability was calculated from the proportion of translated overlaps whose absolute deviation from the reference mean was at least as large as that of the observed overlap, with the observed placement included through the standard + 1/(N + 1) correction.

The excess spatial association between A and B was expressed as a standardised score:

(3) $$z_{AB}\left(r\right)=\frac{J_{AB}\left(r\right)-\langle J_{AB}^{0}\left(r\right)\rangle }{\sigma \left[J_{AB}^{0}\left(r\right)\right]}$$

Here, $\langle J_{AB}^{0}\left(r\right)\rangle$ and $\sigma \left[J_{AB}^{0}\left(r\right)\right]$ are the mean and sample standard deviation of the translated reference distribution. Positive $z_{AB}$ values indicate greater overlap than expected from the occupied areas and internal organisation of the two maps. Negative values indicate lower overlap and spatial separation relative to the same reference. Because the translated distributions need not be Gaussian, zAB was used as a standardised effect descriptor; empirical probabilities were obtained directly from the translation ranks. The ±1.96 interval is used only as a standardised reference band, not as a substitute for the empirical *p* values. The 47,879 translations are dependent realignments of the same two supports, not independent experimental observations. Accordingly, zAB locates the observed overlap within the translation-derived reference distribution, and the empirical probability reports its tail position in that reference rather than a conventional independent-sample significance test.

Robustness at a two-pixel dilation radius was examined in four ways. First, the registered field was divided into four equal quadrants and each quadrant was analysed separately; these values measure internal spatial consistency and were not treated as independent specimens. Second, support B was displaced through all nine combinations of horizontal and vertical shifts from −1 to +1 pixel before analysis. Third, the calculation was repeated after symmetric crop margins of 0, 2, 4, and 6 pixels. Finally, the analysis was repeated with the Dice coefficient to test whether the conclusion depended on the selected overlap metric.

Each binary elemental support was also treated as a scalar field $\phi_{i}\left(x\right)$. Periodic, zero-mean two-point correlations were calculated by fast Fourier transform and normalised by the field variances:

(4) $$C_{ij}\left(r\right)=\frac{\langle \left[\phi_{i}\left(x\right)-\phi_{i}\right]{\left[\phi_{j}\left(x+r\right)-\phi_{j}\right]\rangle }_{x}}{\sigma_{i}\sigma_{j}}$$

Cross-correlations were symmetrised with respect to exchange of i and j before radial averaging. Radial profiles were calculated up to 24% of the smaller image dimension. For each elemental autocorrelation, the effective correlation length $\xi_{i}$ was defined by the first radial distance satisfying

(5) $$C_{ii}\left(\xi_{i}\right)\leq \frac{C_{ii}\left(0\right)}{e}$$

The first zero crossing was also recorded when it occurred within the analysed range. A slowly decaying correlation may contain a contribution from a gradient extending across the image. The resulting values are consequently reported as effective correlation scales of the analysed fields.

### 2.4. Morphological Anisotropy

Directional organisation was quantified in the high-resolution secondary-electron images using a two-dimensional structure tensor [12]. Each original image contained 1280 × 960 pixels. The upper 1280 × 800 pixel region was retained, excluding the scale bar and instrument annotations. The images were converted to greyscale, and their intensities were normalised to the interval [0, 1]. The pixel size determined from the original scale bars was 0.0794 µm for the two 1500× fields.

Before gradient calculation, each image was smoothed with a Gaussian filter having a standard deviation of one pixel. Horizontal and vertical intensity gradients, $g_{x}$ and $g_{y}$, were then calculated with Sobel operators. At a physical analysis scale l, the structure-tensor components were obtained by Gaussian smoothing of the gradient products:

(6) $$J_{l}=\left(\begin{array}{cc} J_{xx} & J_{xy}\\ J_{xy} & J_{yy} \end{array}\right)=\left(\begin{array}{cc} G_{l}*\left(g_{x}^{2}\right) & G_{l}*\left(g_{x}g_{y}\right)\\ G_{l}*\left(g_{x}g_{y}\right) & G_{l}*\left(g_{y}^{2}\right) \end{array}\right)$$

where $G_{l}$ is a Gaussian kernel with a standard deviation of l/p pixels and p is the pixel size in micrometres. Local directional coherence was calculated as

(7) $$c_{l}=\frac{\sqrt{{\left(J_{xx}-J_{yy}\right)}^{2}+4J_{xy}^{2}}}{J_{xx}+J_{yy}+10^{-12}}$$

The term $10^{-12}$ prevents numerical division by zero in regions with negligible gradient energy. Coherence approaches zero when the local gradient energy is distributed similarly in all directions and approaches one when a single direction dominates.

Statistics were restricted to the 40% of pixels with the largest gradient magnitude, selected above the 60th percentile of $\sqrt{g_{x}^{2}+g_{y}^{2}}$. This restriction reduced the influence of nearly uniform image regions in which orientation is poorly defined. Median local coherence was calculated at l=0.2, 0.4, 0.8, and 1.2 µm for the two 1500× fields. The resulting coherence describes directional organisation in the SEM morphology. Crystallographic orientation lies outside this measurement and would require diffraction data.

### 2.5. Computational Implementation

Image processing and statistical analysis were performed in Python 3.12.13 using NumPy 2.3.5, SciPy 1.17.0, pandas 2.2.3, Pillow 12.3.0, and Matplotlib 3.10.8 [13,14,15]. NumPy was used for array operations, exhaustive periodic translations, and Fourier transforms. Binary morphology, Gaussian filtering, and Sobel differentiation were performed with scipy.ndimage. Pillow read the image files, pandas organised the derived numerical data, and Matplotlib produced the graphs.

All stages of the analysis were scripted. Crop coordinates, segmentation thresholds, dilation radii, and physical analysis scales are reported above. Fixed random seeds were used only for the synthetic validation.

### 2.6. Synthetic Validation

The response of $z_{AB}$ to known spatial relationships was examined using synthetic binary fields of 128 × 128 pixels. Clustered scalar fields were generated by applying a Gaussian filter with a standard deviation of 2.2 pixels to normally distributed random values. Each field was converted to a binary support by retaining the upper quantile required to produce an occupied fraction f of 0.05, 0.15, or 0.30.

For each realisation, the first support A was obtained from one clustered random field. Its binary values were smoothed with a two-pixel Gaussian filter to generate an influence field I. A second, independently generated clustered field N supplied the random component of support B. The score used to construct B was

(8) $$S_{B}=\left\{\begin{array}{ll} 1.8I+0.65N, & \mathrm{associated},\\ N, & \mathrm{independent},\\ -1.8I+0.65N, & \mathrm{segregated}. \end{array}\right.$$

The score was thresholded to the same occupied fraction as the first support. Thirty realisations were generated for each combination of the three spatial relationships and three occupied fractions, producing 270 synthetic pairs. Deterministic random seeds depending on occupancy and replicate number were used for both field generation and translation sampling. Each pair was analysed at a one-pixel dilation radius using 199 randomly sampled non-zero toroidal translations. Validation assessed whether associated fields produced positive zAB values, segregated fields produced negative values, and independent fields remained within −1.96 < zAB < 1.96 as occupancy changed.

## 3. Results

### 3.1. Validation of the Spatial Statistic

The synthetic fields were used to determine whether translation normalisation distinguished spatial association from the overlap produced by occupied pixels alone. The statistic returned positive values for every associated pair and negative values for every segregated pair, at occupied fractions of 0.05, 0.15, and 0.30 (Figure S1 and Table S1).

For independently generated pairs, 93.3%, 93.3%, and 100% of the values fell within the interval −1.96 < zAB < 1.96 at occupied fractions of 0.05, 0.15, and 0.30, respectively. Their mean zAB values were 0.18, 0.03, and 0.33. These small positive offsets reflect the finite number and spatial extent of the simulated clusters but remain much smaller than the values obtained for deliberately associated fields.

The raw Jaccard overlap increased markedly with the occupied fraction because denser maps contain more opportunities for coincident pixels. Translation normalisation largely compensated for this dependence: independent fields remained centred close to zero, whereas the sign of the imposed spatial relation was retained. The statistic can therefore be interpreted as excess overlap relative to translations of the same two supports, with their occupancy and internal clustering preserved.

### 3.2. Local Elemental Composition

**Figure 3:**
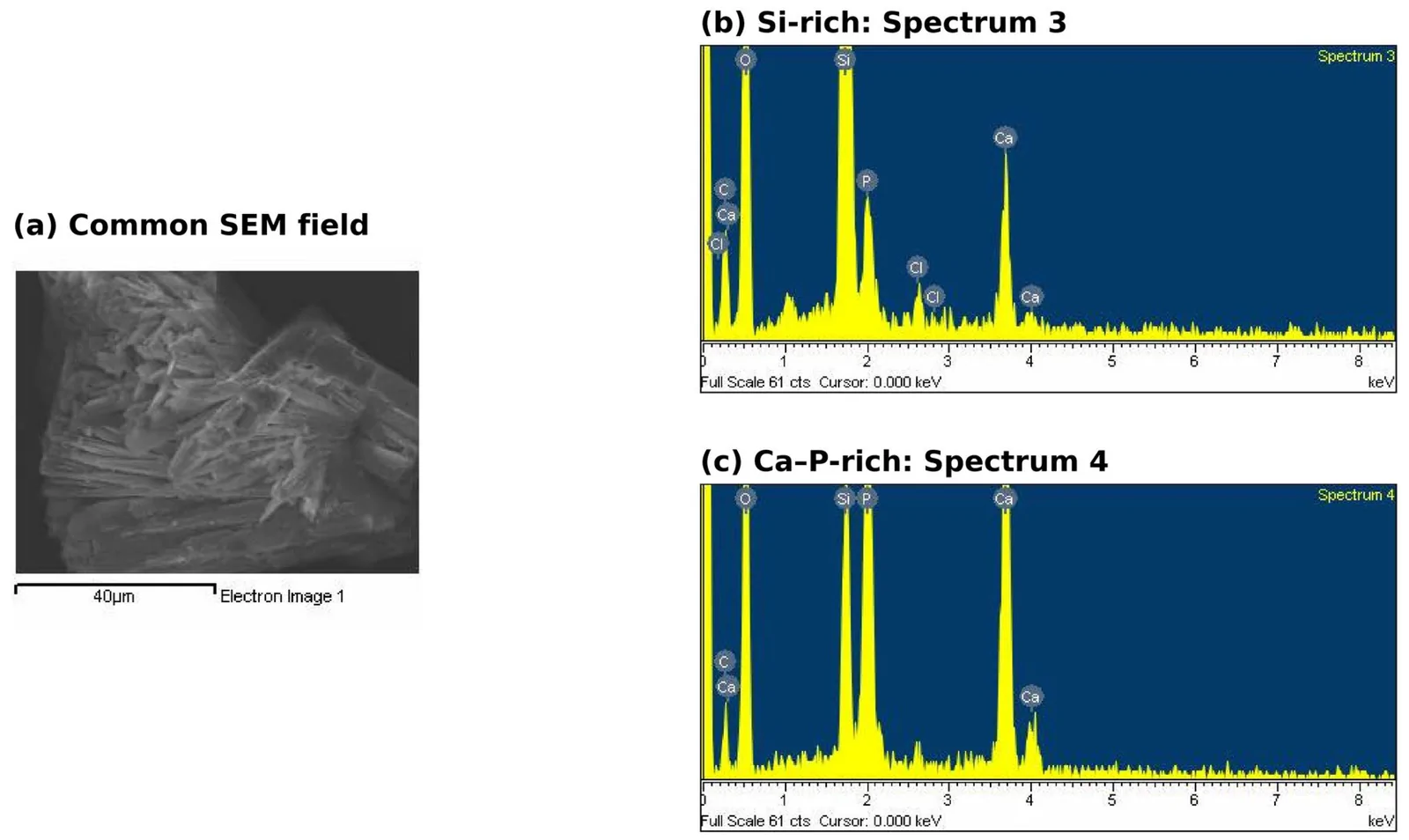
Local SEM–EDS records for 77S-G: (**a**) SEM field reproduced in both original analysis reports; (**b**) Spectrum 3, corresponding to the Si-rich analysis; and (**c**) Spectrum 4, corresponding to the Ca–P-rich analysis. The reports contain identical SEM thumbnails but no probe-marker coordinates. Panel (**a**) identifies the common field without assigning reconstructed acquisition points. Because the exact acquisition positions cannot be reconstructed from the surviving reports, no feature-specific assignment is made.

The 77S-G SEM record shows compact areas alongside aggregates of elongated plates and acicular features. Two local EDS spectra were obtained from the same SEM overview field (Figure 3 and Table 1). Because the exported reports retain no probe-marker coordinates, the spectra cannot be assigned to individual morphological features in the image. The spectra are therefore used only as semiquantitative descriptions of two archived interaction volumes and are not spatially registered to the morphology image or elemental maps.

In the Si-rich analysis, Si represents 25.89 wt.% of the normalised EDS result, while P and Ca account for 2.49 and 2.88 wt.%, respectively. In the Ca–P-rich analysis, the Si contribution decreases to 7.08 wt.%, whereas P and Ca increase to 11.15 and 13.48 wt.%. The measurements therefore distinguish two compositionally different interaction volumes within the same microscopic field.

The Ca/P atomic ratio calculated for the Ca–P-rich interaction volume is 0.93, using the atomic fractions reported by the acquisition software (6.08% Ca and 6.51% P; Table 1). This value is below the stoichiometric Ca/P ratio of 10/6 = 1.67 for hydroxyapatite. The measurement may also include contributions from the underlying silicate because the X-ray interaction volume is not confined to the visible surface feature. The analysed interaction volume is therefore described as Ca–P-rich; the phase identity of the contributing Ca–P-rich material cannot be established from this spectrum.

The Si-rich analysis contains 0.81 wt.% Cl. Because the specimen had been exposed to c-SBF, residual SBF-derived chloride is one possible source, but the archived record does not allow this origin to be established. Carbon occurs in both spectra, at 18.83 wt.% in the Si-rich analysis and 15.14 wt.% in the Ca–P-rich analysis. The archived specimen record does not identify the mounting material, and the spectra provide no basis for separating adventitious carbon, mounting-related carbon, and carbonate incorporated into the reacted surface. Carbon is therefore reported without structural interpretation.

### 3.3. Element-Pair Organisation in the Mapped Fields

The visual overlap between Ca and P is apparent in both sets of elemental maps, although the map occupancies and the clustering of individual signals differ. Translation normalisation provides a reference specific to each field and each extraction setting.

**Figure 4:**
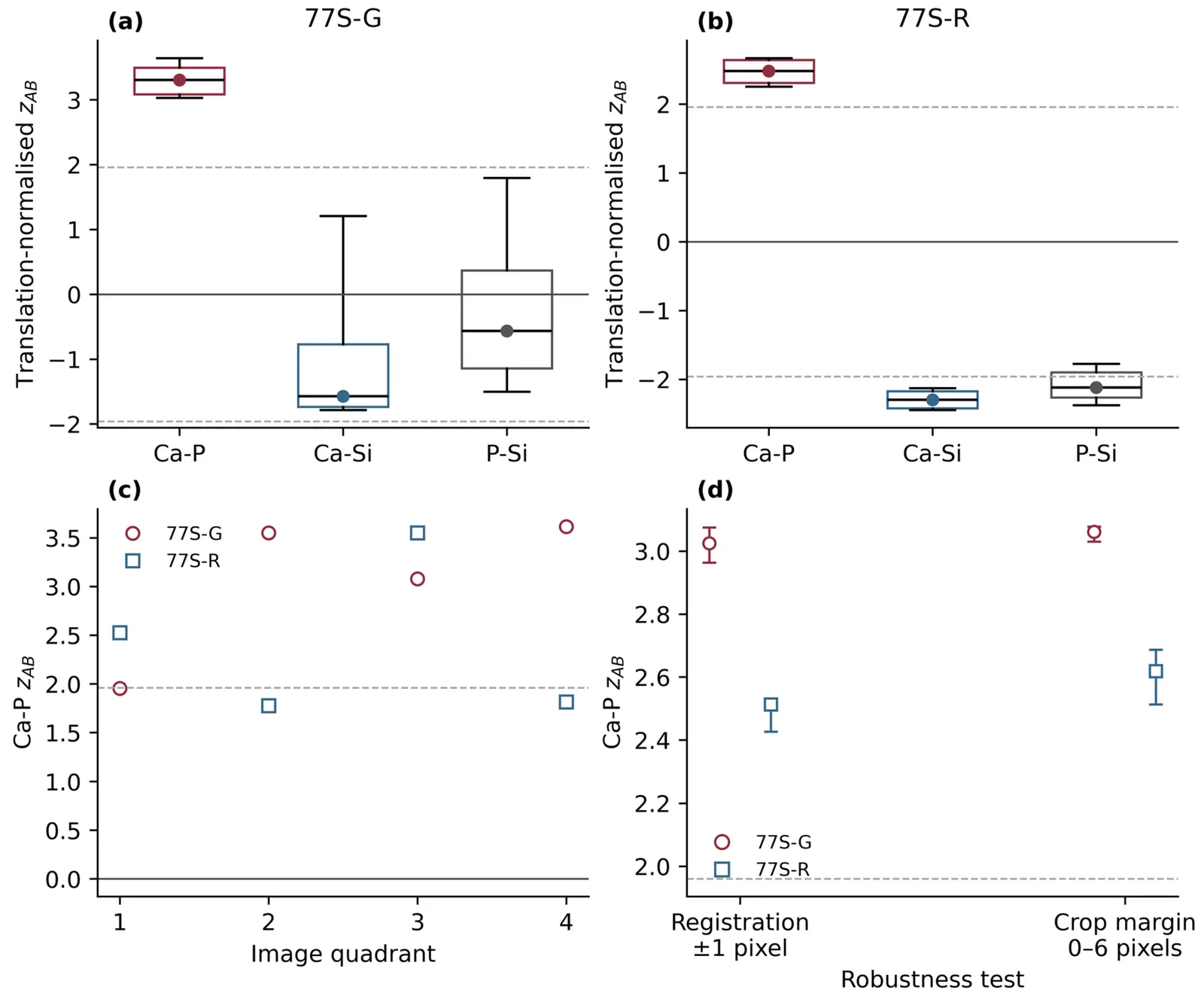
Exhaustive translation analysis at a two-pixel dilation radius. (**a**,**b**) Translation-normalised zAB distributions over the 16 support-extraction settings; boxes span the interquartile range and whiskers the 10th–90th percentiles; filled circles mark the nominal extraction (T = 38, d = 1.22). (**c**) Ca–P values in four image quadrants, used only as an internal-consistency check. (**d**) Median and full range for two robustness tests: nine relative registration offsets spanning ±1 pixel in both directions, and repeated analyses after excluding margins of 0–6 pixels from every image edge. Dashed lines mark z = ±1.96 as a standardised reference band. Quadrants are subfields, not independent specimens.

In 77S-G, the Ca–P association remains positive across all 16 support-extraction settings. At a two-pixel dilation radius (0.32 µm per support), the median translation-normalised zCaP is 3.31, with a full range of 2.99–3.65. For the nominal extraction, zCaP = 3.03 and the two-sided empirical probability is 0.0014. The measured placement of the Ca and P supports therefore produces more overlap than the complete set of non-zero translations of the same spatial patterns (Figure 4a).

The corresponding Ca–Si and P–Si distributions have median values of −1.57 and −0.56, with ranges of −1.80 to 1.24 and −1.50 to 1.80, respectively. Both ranges cross zero. The separation of Ca or P from Si in this field depends on the threshold used to extract the coloured marks and is less stable than the Ca–P association.

In 77S-R, the median translation-normalised zCaP is 2.48 at a two-pixel dilation radius (0.64 µm per support), with a range of 2.09–2.72. The nominal extraction gives zCaP = 2.51 and a two-sided empirical probability of 0.0018. Ca–Si remains below −1.96 in all 16 extraction settings (median −2.30; range −2.50 to −2.06). P–Si is negative in all settings (median −2.12; range −2.54 to −1.48) and exceeds |z| = 1.96 in 11 of 16. The field therefore contains associated Ca and P distributions accompanied by consistent displacement from the displayed Si support, strongest for Ca–Si (Figure 4b).

For both mapped fields, the empirical probabilities describe the position of the observed Ca–P overlap within the translation reference generated from the same two supports; they are not p values derived from independent experimental replicates.

The Ca–P result remained positive under every additional check. It is positive in all four quadrants of each image, with three quadrants above 1.96 in 77S-G and two in 77S-R (Figure 4c). Its range is 2.96–3.08 for the nine ±1-pixel registration perturbations in 77S-G and 2.43–2.53 in 77S-R. Crop margins from 0 to 6 pixels give ranges of 3.03–3.08 and 2.51–2.69, respectively (Figure 4d). Replacing the Jaccard overlap with the Dice overlap preserves the positive, above-threshold association in both fields. These checks establish the stability of the image-derived association descriptor to processing choices within the measured fields, without treating subfields as specimen replication; they do not validate the RGB maps against unavailable quantitative X-ray-count data.

The full 16-setting sensitivity ranges are summarised in Table 2.

### 3.4. Lateral Correlation Scales and Morphological Anisotropy

**Figure 5:**
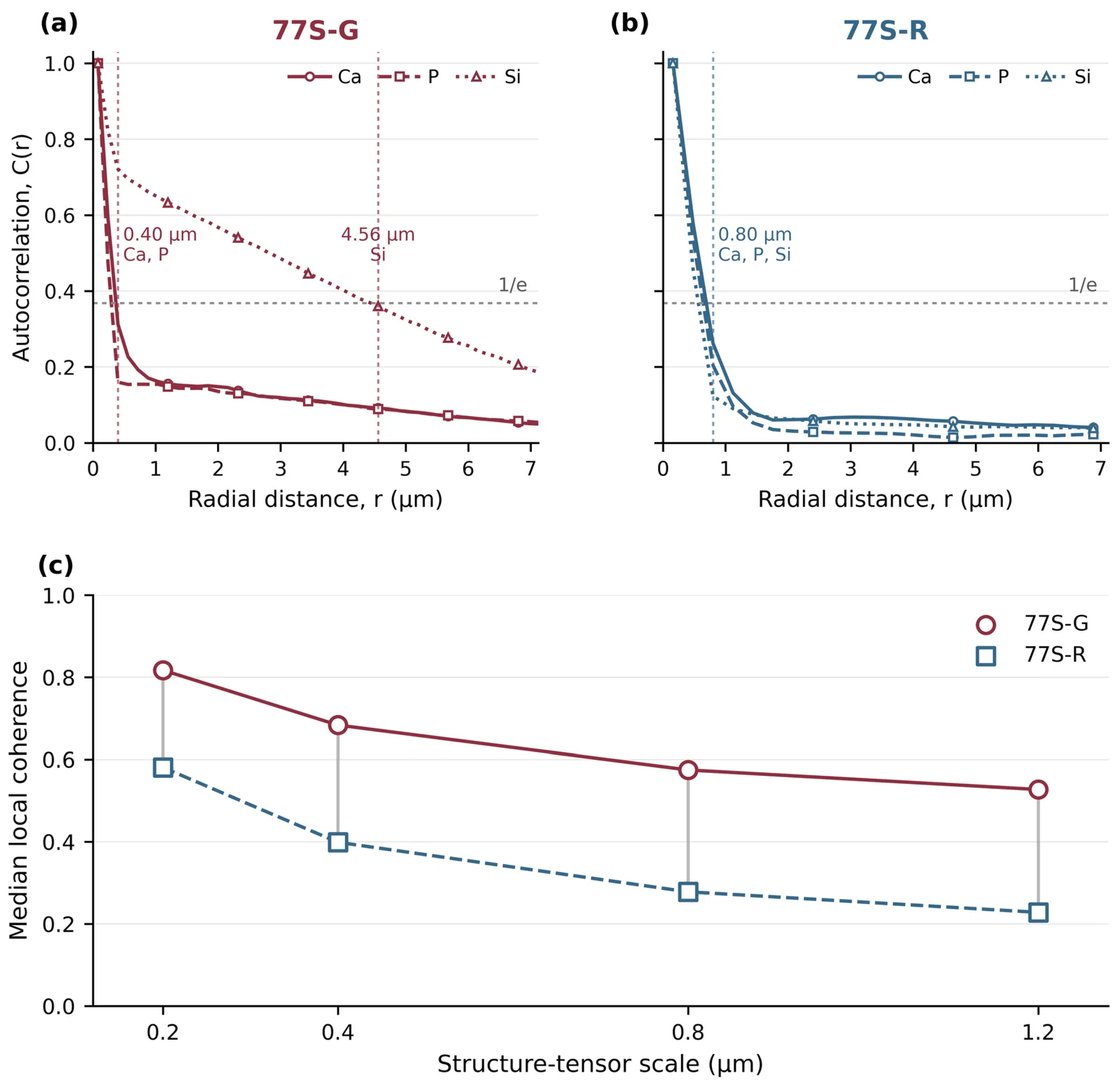
Elemental lateral persistence and morphological directional coherence. (**a**,**b**) Radially averaged autocorrelation profiles C(r) for Ca (solid circles), P (dashed squares), and Si (dotted triangles) in 77S-G and 77S-R, respectively, shown over the common physical range of the two fields. The horizontal line marks 1/e; vertical lines mark the reported effective correlation lengths (77S-G: 0.40 µm for Ca/P and 4.56 µm for Si; 77S-R: 0.80 µm for Ca/P/Si). (**c**) Median local directional coherence at four structure-tensor scales in the 1500× SEM fields. Red open circles: 77S-G; blue open squares: 77S-R; paired values at each scale are connected. Coherence was calculated from the 40% of pixels with the highest gradient magnitude. The reported effective correlation lengths describe field-scale persistence and are not measurements of particle or domain size.

Autocorrelation analysis identifies the distances over which each elemental support retains spatial continuity. In 77S-G, the Ca and P autocorrelations reach C(0)/e at approximately 0.40 µm. Their similar decay scales are consistent with the associated spatial distributions detected by the translation analysis (Figure 5a).

The 77S-G Si autocorrelation decays much more slowly, reaching C(0)/e at 4.56 µm and showing no zero crossing within the analysed range (Figure 5a). The value is therefore controlled by a gradient extending across a substantial part of the image. It is an effective field-scale persistence length and should not be interpreted as the characteristic size of an isolated Si-rich feature.

In 77S-R, the Ca, P, and Si autocorrelations reach C(0)/e at approximately 0.80 µm (Figure 5b). The similar values indicate that the three extracted supports vary over comparable lateral distances, despite the negative Ca–Si and P–Si associations. Correlation length and pairwise association describe different properties: the first measures persistence within each support, while the second measures the relative positioning of two supports.

The structure-tensor analysis provides an independent measure of directional organisation in the SEM texture. At a tensor scale of 0.4 µm, the median local coherence is 0.683 for the 77S-G 1500× field and 0.399 for the 77S-R 1500× field. Within these analysed fields, the 77S-G value remains higher at every scale between 0.2 and 1.2 µm (Figure 5c), consistent with the visually prominent alignment of plates and acicular features in that image.

## 4. Discussion

### 4.1. Elemental Partitioning of the Reaction Front

The central result is not simply that Ca and P occur in the same reacted field, but that their measured relative placement produces more overlap than expected when the original spatial patterns are translated against one another. This distinction is important for reacted bioactive glasses, where both elements may occupy clustered regions over finite areas. In both 77S-G and 77S-R, the positive Ca–P association survives the complete segmentation sweep and the additional registration, crop, quadrant, and overlap-metric checks. The result is therefore consistent with the development of spatially coupled Ca- and P-containing regions described in established models of silicate bioactive-glass reaction and in recent microscopy of reacted glass surfaces [1,2,3]. The present experiment, however, is an endpoint measurement after 216 h and does not establish the temporal sequence by which those regions formed.

The relationship to Si differs between the two mapped fields and is therefore more informative than Ca–P overlap alone. In 77S-R, Ca–Si separation remains below the standardised reference band throughout all 16 extraction settings, while P–Si remains negative and is below −1.96 in most settings. This pattern is consistent with a chemically partitioned reaction front containing Ca–P-rich regions displaced from the displayed Si support, comparable in broad spatial terms with differentiated Ca–P-rich and Si-rich regions reported for reacted 45S5 glass [3]. In 77S-G, by contrast, Ca–Si and P–Si depend on the extraction threshold even though the Ca–P association is stable. The field therefore supports Ca–P coupling but not a threshold-independent statement that the Ca–P-rich population is separated from Si. This difference argues against treating the two reacted surfaces as equivalent simply because both contain Ca and P.

The two local 77S-G spectra add compositional evidence but also define an important boundary on the interpretation. The Ca–P-rich interaction volume has a Ca/P atomic ratio of 0.93, well below the stoichiometric value of 1.67 for hydroxyapatite. Calcium-deficient and non-crystalline Ca–P states can occur during bioactive-glass reaction [3], but the present SEM–EDS spectrum cannot distinguish among amorphous calcium phosphate, calcium-deficient apatitic material, mixtures of reaction products, or contributions from underlying silicate. SEM–EDS is also intrinsically an interaction-volume measurement rather than a phase-specific surface probe [4]. For these reasons, the term “Ca–P-rich” is deliberately retained. The spatial Ca–P association demonstrates coupled elemental organisation; it does not by itself identify hydroxyapatite or any other crystalline phase.

### 4.2. Lateral Organisation and Morphological Order

The autocorrelation results add a length scale to this chemical partitioning (Figure 5a,b). In 77S-G, Ca and P lose correlation over approximately 0.40 µm, whereas the Si support retains a much longer effective persistence of 4.56 µm. The slowly decaying Si profile is dominated by a broad gradient across the field and should therefore be read as field-scale organisation rather than as the diameter of an individual Si-rich feature. In 77S-R, Ca, P, and Si all reach C(0)/e near 0.80 µm. The contrast between the two materials is thus not simply a change in the feature size: the 77S-G field combines short-scale Ca/P organisation with a much more extended Si pattern, whereas the 77S-R supports vary over comparable lateral distances. Earlier image-analysis work on thermally treated bioactive glass quantified changes in particle dimensions and elongation after SBF exposure [7]. The present analysis addresses a complementary spatial question by separating the lateral persistence of elemental supports from directional order in the SEM texture.

Correlation length and pairwise association remain conceptually distinct. Similar autocorrelation lengths do not imply that two elements occupy the same locations: 77S-R provides a direct example, because Ca, P, and Si have comparable effective persistence lengths even though Ca–Si and P–Si associations are negative. Conversely, the similar 0.40 µm Ca and P scales in 77S-G accompany a positive Ca–P association, but the association statistic is required to show that the two supports are positioned together more strongly than expected from their own clustering. Combining the two descriptors therefore separates “how far a pattern persists” from “where one pattern lies relative to another”, avoiding an interpretation based on visual similarity alone.

The structure-tensor result provides a third, independent description of the reacted interface (Figure 5c). The median local coherence is higher in the 77S-G 1500× SEM field at every analysed scale from 0.2 to 1.2 µm, quantitatively supporting the more strongly aligned plate-like and acicular texture visible in that image. Coherence decreases with the increasing analysis scale in both fields, but the separation between them persists across the full range. This multiscale behaviour is more informative than a single orientation value because it shows that the contrast is not confined to one arbitrarily chosen neighbourhood size. At the same time, the SEM field is not pixel-wise registered to the EDS maps, so the aligned morphology cannot be assigned directly to the Ca–P-rich or Si-rich populations. The tensor quantifies texture; it does not establish the chemical identity of the elongated features. Because the complete SEM acquisition conditions were not retained, acquisition-related differences cannot be excluded; the coherence contrast is therefore interpreted only for the displayed fields and not as an intrinsic material property.

### 4.3. Synthesis Route and Interfacial Organisation

The different synthesis media provide context for comparing 77S-G and 77S-R, but they are not variables in the image-analysis workflow. In related sol–gel bioactive-glass systems, the replacement of ultrapure water by Azorean geothermal waters altered the surface chemical environment and silicate-network connectivity detected by XPS [9]. The present study does not test the composition of the synthesis water and cannot associate any reported spatial descriptor with a particular dissolved component. It therefore reports only the field-level contrast between the archived microscopy records.

That comparison should nevertheless remain cautious. The previous XPS study examined related, not identical, glass compositions, and the present experiment does not isolate individual geothermal-water constituents or provide independent synthesis-batch replication. The current results therefore do not establish a causal link between a particular dissolved ion and the observed micrometre-scale morphology. They document a field-level difference between the archived records without identifying its origin. More generally, the 77 mol% SiO_2_ sol–gel composition studied here is not a direct analogue of 45S5, and recent work has emphasised that the details of silica-rich-layer development and the development of Ca–P-rich regions can depend strongly on the glass composition and reaction conditions [2,3]. The classical reaction sequence is consequently used here as mechanistic context rather than as a phase-identification template.

### 4.4. Analytical Scope and Limitations

A methodological advantage of the translation analysis is that it tests relative elemental placement while preserving the occupied area, shape, and internal clustering of each measured support. Because the statistic operates on binary geometry rather than display intensity, it does not reinterpret JPEG colour values as elemental concentration. Quantitative SEM–EDS studies increasingly seek to move beyond qualitative map juxtaposition by treating spatial colocation explicitly [5,6]. For bioactive-glass characterisation, the present approach adds a computational layer to experimental mapping while preserving what the archived occurrence maps actually contain. It addresses a specific difficulty of clustered maps: a high raw overlap can arise simply because two supports are extensive or internally aggregated. The complete toroidal reference asks a narrower question—whether the observed placement is exceptional relative to all other placements of the same two patterns. Synthetic associated, independent, and segregated fields, together with the 16 extraction settings and the registration, crop, quadrant, and Dice checks, show that the Ca–P conclusion is not tied to a single threshold or overlap definition. The methodological context in references [4,5,6,7] distinguishes the quantitative SEM–EDS reliability, elemental-colocation analysis, and prior image analysis of SBF-reacted bioactive glass from the present translation-based spatial reference.

The strength of that inference is nevertheless bounded by the archived data. The elemental maps are 8-bit RGB JPEG occurrence maps rather than raw X-ray count maps, so the analysis quantifies spatial geometry and not pixel-resolved concentration [4]. The toroidal reference imposes periodic boundary conditions at the field edges; the crop-margin analysis reduces the sensitivity to edge content but does not remove this modelling convention. The morphology images and EDS maps are separate acquisitions, and only one 1500× SEM field and one mapped EDS field were analysed for each material. The 77S-G/77S-R differences must therefore remain field-level contrasts rather than estimates of specimen-wide prevalence. Additional independently acquired fields, preferably retaining raw-count EDS data and co-registered SEM morphology, would allow the same framework to test between-field reproducibility and direct morphology–chemistry coupling. Within the available fields, however, the exhaustive translation reference and the multiple sensitivity checks provide a well-constrained basis for the principal conclusion: Ca and P are spatially associated in both reacted surfaces, while their relationship to Si and the directional organisation of the morphology differ between the two analysed materials. Because the complete detector configuration and acquisition parameters were not retained, differences in the SEM texture or map appearance may also contain acquisition-related contributions; this is an additional reason not to treat the field contrasts as intrinsic material properties. Independent X-ray diffraction and/or Fourier-transform infrared spectroscopy would provide complementary phase/structural characterisation. These measurements are not available for the archived dataset; accordingly, the present work deliberately restricts its conclusions to image-derived spatial organisation and does not assign a crystalline phase. Such acquisitions would constitute an independent validation dataset rather than a retrospective correction of the experimental records analysed here. Within this restricted evidential scope, the retained experimental records are sufficient to support the reported field-level spatial descriptors: the analysis does not require pixel-resolved concentration to test the relative support placement, lateral persistence, or directional coherence. The stability of the principal Ca–P result across the segmentation sweep and the additional registration, crop, quadrant, and overlap-metric checks shows that the reported spatial conclusion is not contingent on a single image-processing choice. The methodological contribution is therefore the demonstration that spatial information retained in conventional SEM–EDS occurrence-map exports can be quantified beyond qualitative visual comparison, provided that interpretation is confined to the information actually preserved in the maps and accompanied by explicit sensitivity testing. New acquisitions could extend the dataset and independently validate the workflow across additional fields, but they are not required for the validity of the field-level conclusions reported here.

## 5. Conclusions

Within the analysed fields, the reacted interfaces show micrometre-scale chemical and morphological organisation. Ca and P form spatially associated distributions in both mapped EDS fields. In 77S-R, their positions are also consistently displaced from the Si support, most strongly for Ca–Si. In 77S-G, the maps establish Ca–P association but not threshold-stable separation from Si; the two local EDS analyses independently show Si-rich and Ca–P-rich interaction volumes.

The field-preserving translation reference shows that the measured Ca–P coincidence exceeds the overlap generated by translations that preserve occupancy and internal clustering. The association remains positive across all 16 segmentation settings and through the quadrant, registration, crop-margin, and overlap-metric checks. Synthetic fields recover the imposed associated, independent, and segregated arrangements across three occupied fractions, providing a controlled validation of the statistic.

Morphology provides a complementary result. The 77S-G 1500× field has higher directional coherence than the 77S-R 1500× field across structure-tensor scales from 0.2 to 1.2 µm, while elemental autocorrelations identify distinct persistence scales within the mapped reaction fronts. The framework therefore provides three field-level descriptors—elemental association, effective lateral correlation length, and morphological anisotropy—that can be applied to other heterogeneous interfaces when exported maps preserve spatial registration. Used in this role, computational spatial analysis complements rather than replaces phase-specific spectroscopy or diffraction and replicated microscopy. Specimen-wide prevalence would require additional independently acquired fields. Within this defined scope, the present experimental records are sufficient to establish the reported field-level descriptors and to demonstrate the applicability of the workflow to heterogeneous reacted bioactive-glass interfaces. Additional independently acquired fields would test broader reproducibility and specimen-wide prevalence, rather than determine whether the spatial descriptors reported for the analysed fields are valid.

## Figures and Tables

**Table 1:** Normalised local EDS results supplied by the acquisition software. Values are given as wt.% with atomic % in parentheses and refer to the selected interaction volumes. Carbon may include mounting material or surface contamination.

Region	C	O	Si	P	Ca	Cl
Si-rich analysis	18.83 (27.35)	49.09 (53.52)	25.89 (16.08)	2.49 (1.40)	2.88 (1.25)	0.81 (0.40)
Ca–P-rich analysis	15.14 (22.79)	53.15 (60.06)	7.08 (4.56)	11.15 (6.51)	13.48 (6.08)	—

**Table 2:** Sensitivity of the spatial association to 16 map-extraction settings at a two-pixel dilation radius. Brackets give the full range across the 16 settings.

Field	Pair	r (µm)	Median z [Range]	Interpretation
77S-G	Ca–P	0.32	3.31 [2.99, 3.65]	association
77S-G	Ca–Si	0.32	−1.57 [−1.80, 1.24]	threshold-dependent
77S-G	P–Si	0.32	−0.56 [−1.50, 1.80]	threshold-dependent
77S-R	Ca–P	0.64	2.48 [2.09, 2.72]	association
77S-R	Ca–Si	0.64	−2.30 [−2.50, −2.06]	separation
77S-R	P–Si	0.64	−2.12 [−2.54, −1.48]	separation

## Data Availability

The numerical results supporting the central analyses are reported in the article and Supporting Information. The analysis code and numerical output tables used in the present work are provided as Online Resource 2.

## Supplementary Materials

The following supporting information can be downloaded at: https://www.mdpi.com/article/10.3390/ma19204317/s1, Figure S1: Synthetic validation; Table S1: Synthetic-validation summary; Table S2: exhaustive nominal-field results; Table S3: Ca–P robustness ranges; Figure S2: nominal binary elemental supports. Online Resource 2 contains the analysis code and numerical output tables documenting the reported image-derived calculations.

## Abbreviations

c-SBF, corrected simulated body fluid; EDS, energy-dispersive X-ray spectroscopy; FTIR, Fourier-transform infrared spectroscopy; JPEG, Joint Photographic Experts Group; RGB, red–green–blue; SBF, simulated body fluid; SEM, scanning electron microscopy; XPS, X-ray photoelectron spectroscopy; XRD, X-ray diffraction.
