# Supplementary material for: Micrometre-Scale Surface Partitioning and Morphological Anisotropy in Reacted Bioactive Glass Quantified by SEM–EDS
Source: Materials (Basel). 2026 Oct 14;19(20):4317. doi: 10.3390/ma19204317 (PMC13707233; doi:10.3390/ma19204317)
Supplement: Supplementary file 1 [file materials-19-04317-s001.zip › Supporting_Information.pdf]

Supporting Information

Micrometre-scale surface partitioning and morphological anisotropy in reacted bioactive glass quantified by SEM–EDS

Helena Cristina Vasconcelos[1,2*] and Maria Gabriela Meirelles[1,3]

[1] Faculty of Science and Technology, University of the Azores, S. Miguel, Azores, 9500-321 Ponta Delgada, Portugal

[2] Laboratory of Instrumentation, Biomedical Engineering and Radiation Physics (LIBPhys, UNL), Department of Physics, NOVA School of Science and Technology, 2829-516 Caparica, Portugal

[3] Research Institute of Marine Sciences, University of the Azores (OKEANOS), Faial, Azores, 9901-862 Horta, Portugal

* Correspondence: helena.cs.vasconcelos@uac.pt

Figure S1. Synthetic validation

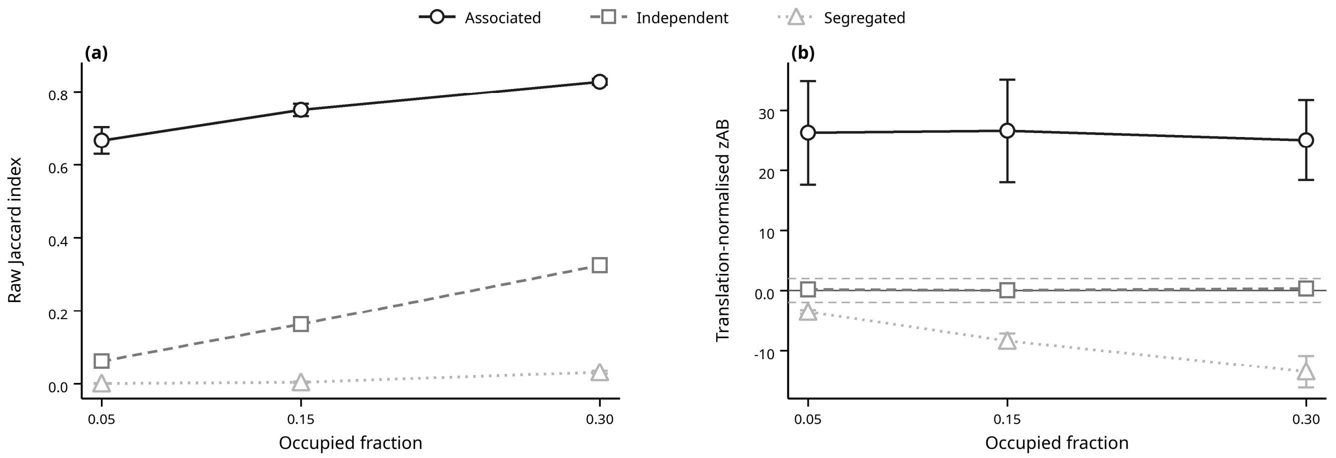

Figure S1. Synthetic validation across occupied fractions of 0.05, 0.15 and 0.30: (a) raw Jaccard overlap and (b) translation-normalised zAB for associated, independent and segregated fields. Each condition contains 30 realisations. Points are means and error bars show ±1 standard deviation across realisations.

Table S1. Synthetic-validation summary

Relation	Occupancy	n	Mean zAB	Within ±1.96 (%)
associated	0.05	30	26.30	—
independent	0.05	30	0.18	93.3
segregated	0.05	30	−3.55	—
associated	0.15	30	26.62	—
independent	0.15	30	0.03	93.3
segregated	0.15	30	−8.37	—
associated	0.30	30	25.07	—
independent	0.30	30	0.33	100.0
segregated	0.30	30	−13.51	—

Table S2. Exhaustive nominal-field results

Nominal extraction: brightness threshold T = 38 and channel-dominance factor d = 1.22. The reference comprises all 47 879 non-zero toroidal translations of the same pair of dilated supports.

Field	Pair	r (µm)	Observed J	Reference mean J	zAB	Two-sided p
77S-G	Ca–P	0.32	0.5700	0.4433	3.030	0.00142
77S-G	Ca–Si	0.32	0.2427	0.3494	−1.427	0.1772
77S-G	P–Si	0.32	0.4242	0.4288	−0.107	0.9218
77S-R	Ca–P	0.64	0.4352	0.3594	2.513	0.001754
77S-R	Ca–Si	0.64	0.4454	0.5032	−2.465	0.004678
77S-R	P–Si	0.64	0.3252	0.3677	−2.107	0.01324

Table S3. Ca–P robustness ranges

Field	Check	Placements	Median zAB	Full range
77S-G	Quadrants	4	3.316	1.953–3.616
77S-G	Registration ±1 pixel	9	3.025	2.964–3.075
77S-G	Crop margin 0–6 pixels	4	3.061	3.030–3.078
77S-R	Quadrants	4	2.171	1.777–3.552
77S-R	Registration ±1 pixel	9	2.513	2.426–2.529
77S-R	Crop margin 0–6 pixels	4	2.619	2.513–2.687

Quadrant values are internal-consistency checks only and are not independent specimen replicates.

Dice gave zAB = 2.868 (77S-G) and 2.415 (77S-R), with two-sided empirical p = 0.00142 and 0.00175, respectively.

Figure S2. Nominal binary elemental supports

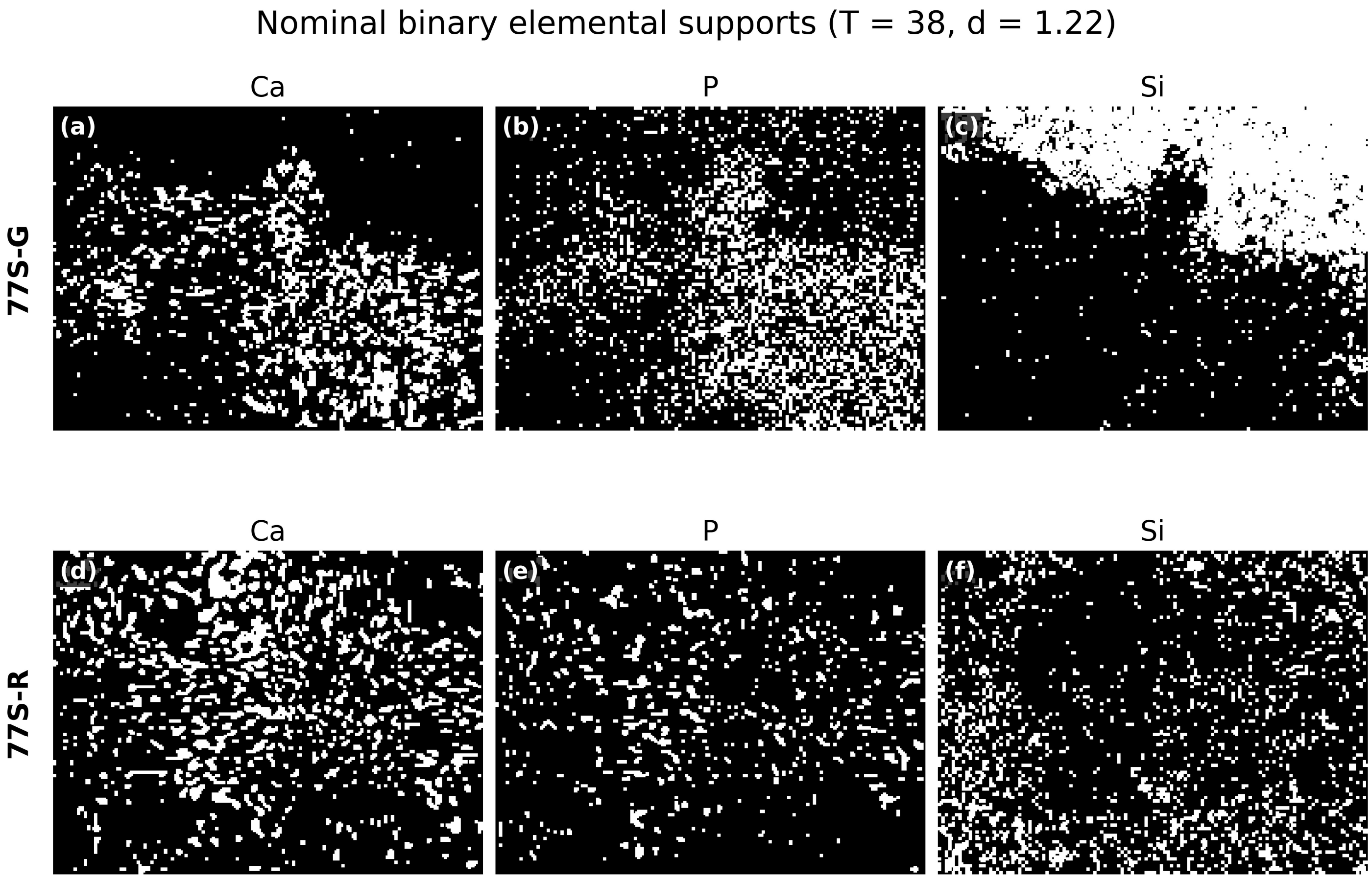

Figure S2. Binary Ca, P and Si supports obtained with the nominal extraction setting (T = 38, d = 1.22) after the 2 × 2 pixel binary opening and before dilation. Panels (a–c) show 77S-G and panels (d–f) show 77S-R. White pixels denote the retained elemental support. The pixel sizes of the analysed fields are 0.16 μm for 77S-G and 0.32 μm for 77S-R.
